# A New Technique of Brachioradialis Tendon Rerouting Combined With Osteotomy in Contractures of the Forearm

**DOI:** 10.1097/BTH.0000000000000522

**Published:** 2025-06-05

**Authors:** Ladislav Nagy, Tudor Trache, Lisa Reissner

**Affiliations:** Department of Hand Surgery, Balgrist University Hospital, Zurich, Switzerland

**Keywords:** brachioradialis rerouting, forearm osteotomy, obstetric brachial plexus palsy

## Abstract

Rotational deformities of the forearm occur due to neurological imbalance of pro/supinating forces, commonly seen in brachial plexus birth injury. Brachioradialis rerouting has been described for the correction of active pronation or supination deficits. Fixed deformities cannot be addressed by soft tissue procedures alone, and need derotational osteotomies. More recently, techniques combining biceps rerouting and forearm osteotomy have been described. We present an alternative technique to correct fixed rotational forearm deformities by derotational forearm osteotomy and brachioradialis tendon rerouting. The technique can be used to correct both supination and pronation deformities, because the brachioradialis tendon can be rerouted to support either of the 2. No tenotomy and no tendon reconstruction has to be performed. This allows for early mobilization and eliminates the risk of complications at the site of tendon reconstruction.

Rotational deformities of the forearm occur as an effect of neurological deficits with prolonged imbalance of pronation/supination forces, commonly encountered in brachial plexus birth injury (BPBI).^1–3^ Even though BPBI patients are traditionally described to have supination deficits, more recent studies showed supination deformities of the forearm (“begging hand”) to be more prevalent and more disabling in the long term, as pronation of the forearm is more relevant for everyday activities than supination.^4–6^ A minimum of 30 degrees of pronation are required for activities of daily living.^7^

In an early report, Steindler^8^ documented satisfactory correction only by release of the supinator and biceps muscles. Blount highlighted that osseous deformities developed over time, rendering standalone soft tissue procedures as ineffective. He advocated osteoclasis for osseous correction.^9^ Grilli^10^ brought about the concept of rerouting the biceps brachii into a pronating muscle. Forearm osteotomy has been validated for treatment of supination deformities, but recurrence rates of up to 40% have been reported.^1,5,11–13^ Zancolli^14^ established the biceps brachii rerouting as an alternative procedure to counteract excessive supination forces and improve active pronation. The technique combines the rerouting of the distal biceps tendon around the proximal radius with the release of the interosseous membrane. There is general consensus that irreducible/fixed contractures cannot be addressed by biceps rerouting alone but must be combined with corrective—derotational osteotomies or, less recommended, a release of the interosseous membrane.^2,15,16^ More recently, combined procedures of osteotomy and biceps tendon rerouting, and also alternative reinsertion methods of the rerouted biceps tendon have been described, promising improved biomechanical properties.^17^ Nevertheless, biceps rerouting is not recommended for cases with paralysis of elbow extension and/or subluxation of the radial head.^18^

Özkan et al^19,20^ described an alternative technique of improving forearm rotation in BPBI, by rerouting the brachioradialis (BR) tendon. This technique does not have the contraindications of biceps rerouting and is suitable for treating both supination and pronation deficits, depending on the rerouting direction relative to the forearm rotation axis. The technique requires a Z-shaped tenotomy, rerouting of the tendon ends around the distal radius, and reattachment though a and Pulvertaft weave suture. The arm is immobilized in an above-elbow cast, with the elbow at 90 degrees, for 3 to 4 weeks postoperatively.

The senior author of the preset study has modified the technique of brachioradialis rerouting when combined with corrective osteotomy of the radius in patients with fixed rotational deformities of the forearm, which does not imply a tenotomy and subsequent tendon reattachment.

## ANATOMY

The brachioradialis muscle is innervated by the radial nerve (C5 to C7) and has its origin on the lateral edge of the distal third of the humerus and on the lateral intermuscular septum. It inserts on the radial styloid. It functions as an elbow flexor in neutral forearm rotation, as a supinator in pronation and as a pronator in supination. Biomechanical studies have shown it to be the only muscle offering significant pronation support in extreme supination.^21^ The course of its tendon close to the radial diaphysis allows for rerouting through the same approach used for radius osteotomy. Our technique makes use of this proximity, rerouting the brachioradialis tendon through the osteotomy and eliminating the need for tenotomy and tendon repair.

## INDICATIONS/CONTRAINDICATIONS

The patients present with paralytic rotational deformity of the forearm without a bony reason for the restriction. The deformity is totally or partially restricting the range of motion and especially not allowing for adapting a functional position even by passive manipulation. Typically, the residual muscle function does not suffice to exhaust the passive range of motion. We performed the technique in 3 patients. Two presented with recurrent supination deformity due to BPBI and one with a pronation deformity. In all patients, the deformity induced relevant impairment of hand function in activities of daily living. Bony deformity and radial dislocation were radiologically excluded. All had massive weakness of elbow extension strength, 2 had an extension deficit. Written informed consent was obtained from all patients before the study.

## TECHNIQUE

### Setup

Preoperative analysis of the deformity was performed, and the extent of osseous derotation was determined. This amount was at least as much as the passive pronation deficit.

The patient was placed supine on the operating table. The procedure can be performed in brachial plexus anesthesia or in general anesthesia. A tourniquet was placed around the upper arm. The upper extremity was prepped and draped, then exsanguinated and the tourniquet was inflated.

### Exposure/Reconstruction

The procedure was performed through a dorsoradial approach. The interval between the brachioradialis and the extensor carpi radialis longus was carefully dissected and the superficial branch of the radial nerve was identified and protected throughout the operation. The radius diaphysis was exposed at this level and the brachioradialis tendon was fully mobilized.

**FIGURE 1:**
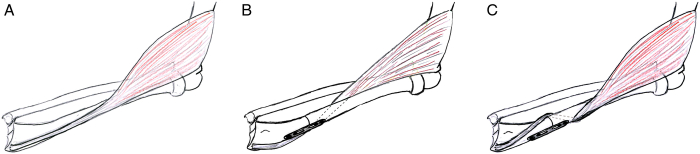
(A) Right forearm dorsal: course of the brachioradialis (BR) muscle and tendon. (B) Right forearm dorsal: rerouting of the BP tendon to aid pronation. (C) Right forearm dorsal: rerouting of the BP tendon to aid supination.

**FIGURE 2:**
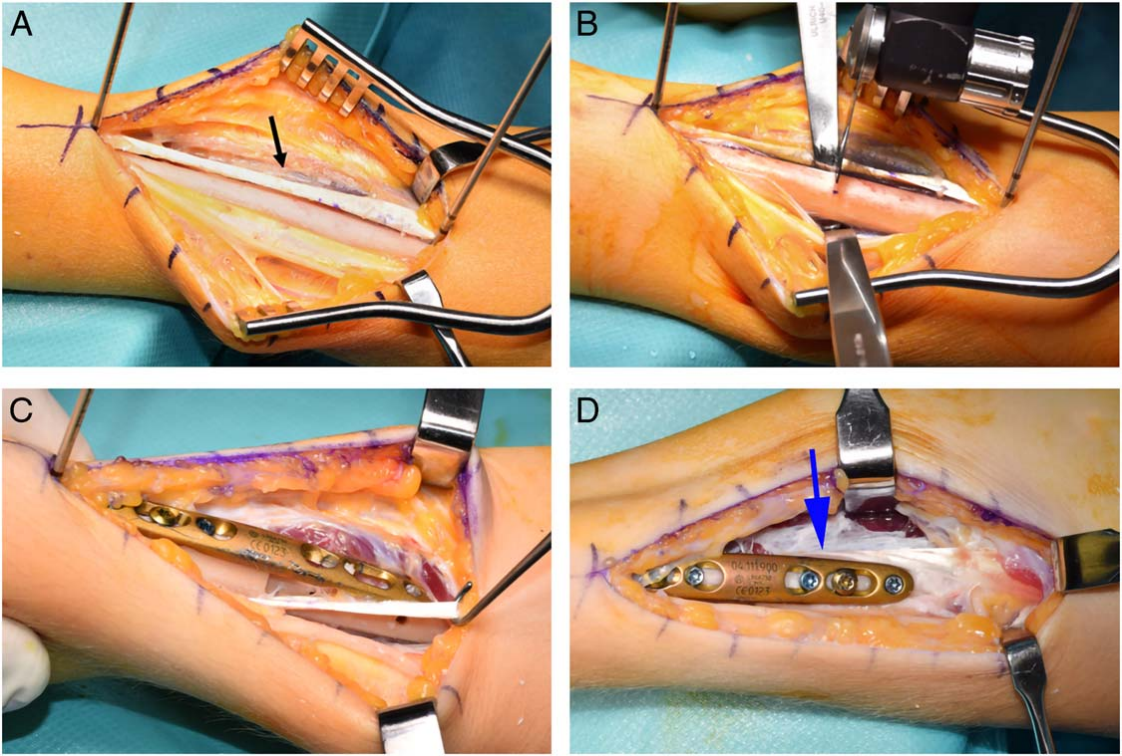
(A) Left distal forearm: mobilized brachioradialis (BR) tendon (black arrow). (B) Left distal forearm: osteotomy. The BR tendon can then be passed through the osteotomy to either side of the radius, according to the intended function (pronation/supination). (C) Left distal forearm: BR tendon rerouted through the osteotomy towards dorsal to aid pronation. (D) Left distal forearm. BR tendon rerouted through the osteotomy volar to aid supination (blue arrow: volar position of the BR in the volar direction).

The correction was planned at the level of the distal third of the radius diaphysis (Figs. 1A, 2A, B). The position of the plate for subsequent fixation was defined and the rotational positions of the distal and proximal fragments were marked with Kirschner wires. The adequately sized plate (6 or more holes) was placed aligned and provisionally fixed on the distal fragment with 2 screws, then removed. Hereafter, a transverse osteotomy of the radius was performed. For a pronating effect, the proximal radius was passed over the BR tendon (Fig. 2C). This results in a new, spiral course of the brachialis tendon, very much like a dorsal prolongation of the pronator teres tendon. Naturally, this longer course of the tendon results in an increase of its tension. This; however, was released by the rotational corrective osteotomy which follows. The plate was fixed back again to the distal fragment which now can be pronated as much as necessary in order to compensate the fixed pronation deficit (Fig. 1B). The goal was a neutral or slightly pronated position at rest with at least 30 degrees of additional passive pronation. To achieve this, the free part of the plate was clamped to the proximal fragment allowing for testing of the new position. Once this was achieved, the plate was definitively fixed to the proximal radius fragment. Eccentric placement of the screws permitted optimal compression of the osteotomy and a stable fixation.

To correct a fixed pronation contracture, the proximal radius was passed under the BR tendon resulting in a spiral course parallel to the muscles of the first extensor compartment (Fig. 1C). The rotational correctional osteotomy occurs in the way described above, obviously in the opposite direction, supinating the distal fragment (Fig. 2D).

### Closure

The tourniquet was deflated and hemostasis was performed. The wound was thoroughly irrigated and a layered closure was performed. The use of a drain tube may be indicated. The extremity was placed in a protective removable forearm splint.

### Rehabilitation

A protective removable splint was used for 6 to 8 weeks postoperatively according the documented consolidation of the osteotomy. Unloaded mobilization of the wrist and forearm under ergotherapeutic monitoring was allowed as soon as possible. After the evidence of consolidation, splinting was discontinued and full weight bearing and use of the hand was allowed.

## EXPECTED OUTCOMES

The passive rotational deficit of the forearm was corrected in all cases. Patient 1 improved his active pronation from 0 to +60 degrees. Patient 2 improved her active pronation from −30 to +40 degrees. Patient 3 had a pronation deformity with active range of motion of 90/70/0 degrees, which was corrected to 90/20/0 degrees, more suitable for the activities of daily living. Table 1 depicts the postoperative evolution of the passive and active ROM in our patients.

**TABLE 1:** Active and Passive ROM Preoperative and Postoperative

	Preoperative active pronation/supination (deg.)	Passive pronation/supination (deg.)	Postoperative active pronation/supination (deg.)	Passive pronation/supination (deg.)
Patient 1	0/0/65	30/0/100	60/0/10	90/0/40
Patient 2	0/30/70	0/0/90	40/0/20	50/0/50
Patient 3	90/70/0	120/30/0	90/20/0	105/0/10

The deforming forces leading to pro-/supination deformity are opposed by the pull of the rerouted brachioradialis, leading to a reduction of the deformity—generating forces. Therefore, a lower recurrence rate of the deformity can be expected for combined corrective osteotomy and BR rerouting. No recurrence was observed in our patients after a maximum follow-up of 14 months. Nevertheless, longer observation periods are required to verify this expectation. Ideally, rerouting the BR muscle pull to a pronating/supinating function would also improve the active range of motion, providing the muscle is properly innervated.

Supination deformity of the forearm is a disabling and disfiguring condition than can follow obstetric paralysis or other paralytic conditions. There is a general agreement, that if the deformity is easily reducible, a dynamic rebalancing procedure such as a tendon transfer is appropriate. If, however, the desired range of motion or functional position cannot be obtained by passive manipulation, a corrective rotational osteotomy of the forearm needs to performed. The alternative, a release of the interosseous membrane, is considered as overly invasive and less reliable. Recent literature describes superior outcomes regarding recurrence of deformity and revision rates for combined procedures of forearm osteotomy and biceps rerouting.^18^ Combining static correction of a deformity with a procedure which counteracts the forces leading to that deformity, thus appears the most promising approach.

This is the first description of a combined technique of radius osteotomy and brachioradialis rerouting for correction of rotational forearm deformities. The brachioradialis tendon was rerouted through the osteotomy and not tenotomized. Besides its function as an elbow flexor, the brachioradialis can act as a pronator or as a supinator, depending on the position of the forearm. With the forearm in full supination, it has been shown to be the only muscle to provide significant pronating potential.^21^ With the described technique this potential can be markedly increased or reversed according to the functional demand. As in this technique the BR-tendon is not severed and sutured, stretching or rupture of the tendon cannot occur and functional aftertreatment, especially forearm rotation can be initiated instantly and the elbow does not need any immobilization or restrictions. Therefore, the risk of worsening the function of elbow and/or wrist flexion/extension may be minimized and training of the new function of the BR muscle can be commenced early, maximizing rehabilitation potential.

The main limitation of the present study is the small patient collective and a relatively short follow-up time. Furthermore, the functional outcome of the rerouted brachioradialis (aiding active pro/supination) is probably determined by the preoperative innervation and functional state of this muscle. While adequate preoperative contractility and innervation of the brachioradialis were observed clinically, no electromyographic studies were performed. Therefore, the interdependency between preoperative muscle function and active postoperative pro/supination could not be quantified.

## COMPLICATIONS

No surgical complications occurred, and the osteotomy consolidated in all patients. The procedure has the same complication potential as any standalone corrective osteotomy of the forearm, for which nonunions with hardware failure have been described.^1^
